# Depression and Coping Styles of College Students in China During COVID-19 Pandemic: A Systemic Review and Meta-Analysis

**DOI:** 10.3389/fpubh.2021.613321

**Published:** 2021-07-07

**Authors:** Shengyu Guo, Atipatsa Chiwanda Kaminga, Jie Xiong

**Affiliations:** ^1^Department of Economics and Management, Changsha University, Changsha, China; ^2^Department of Mathematics and Statistics, Mzuzu University, Mzuzu, Malawi; ^3^Department of Mathematics and Computer Science, Changsha University, Changsha, China

**Keywords:** COVID-19, depression, college students, meta-analysis, mental health apps

## Abstract

**Background:** The rapid spread and uncertain outcome of the 2019 novel coronavirus disease (COVID-19) around the world have caused worry, fear, and stress among the general population. Nevertheless, the prevalence of depression among college students in China during lockdown, following the COVID-19 pandemic, and their coping strategies have not been quantitatively assessed.

**Objective:** We aimed to evaluate the prevalence of depression among college students in China during the lockdown due to the COVID-19 pandemic and assess their coping strategies.

**Methods:** Systematic review and meta-analysis were conducted to assess the prevalence of depression among college students in China and their coping strategies.

**Results:** The results indicated that, during lockdown in the COVID-19 pandemic, the prevalence rates of college students in China suffering from mild, moderate, and severe depression were 25% (95% CI = 17–33%), 7% (95% CI = 2–14%), and 2% (95% CI = 1–5%), respectively. Besides, the proportion of college students who use WeChat and Weibo to acquire COVID-19 knowledge was 39% (95% CI = 13–68%), whereas the proportion of college students using mental health application services (APPs) to deal with depression was 59% (95% CI = 41–73%).

**Conclusions:** The prevalence of depression among college students in China was high during the lockdown in the COVID-19 pandemic. Thus, considering the adverse outcomes of depression, it is imperative to screen college students in China for depression during the CIVID-19 pandemic and provide them with necessary psychological interventions to control and prevent depression. Social media platforms, such as WeChat and Weibo, and mental health APPs could provide an opportunity for psychological health information dissemination for college students. However, their effectiveness in reducing depression will have to be assessed.

## Introduction

Coronavirus disease (COVID-19) started in Wuhan, one of the biggest cities in China, in 2019 and quickly spread to almost every human settlement on the planet. The outbreak has been particularly severe in the United States, India, and Brazil such that each had more than 2 million cases. According to a report of the World Health Organization (WHO), there were nearly 20 million COVID-19 patients all over the world, and about three-quarters of a million people died from this disease. Although millions of patients have recovered from the disease, their quality of life has been severely affected by different side effects (1). Furthermore, public health measures including social restrictions and quarantines have been adopted by countries to control the spread of COVID-19, and these have also seriously affected the lives of billions of people (2). Some studies have indicated that the outbreak of COVID-19 and the social isolation policies adopted by countries could lead to serious mental health problems in the general population (3, 4).

Specifically, college students in China were seriously affected by the outbreak of COVID-19 in that most of them were confined to the same place during lockdowns, and emerging data have suggested that the COVID-19 pandemic has brought unbearable psychological pressure to many people (5), including college students (6). For example, some studies have confirmed that COVID-19 has caused an increase in anxiety and depression among medical staff (7), while other studies have found that socially isolated college students have higher rates of unhealthy behaviors, such as longer cell phone use (8) and smoking and drinking (9, 10). However, these studies did not quantitatively evaluate the mental health problems of college students amidst the COVID-19 pandemic and the related prevention and control measures. Therefore, quantitative studies, such as meta-analysis, that can provide more valuable information for the improvement of mental health services in colleges are warranted.

Clinically, depression can be classified into mild, moderate, and severe according to the symptoms of the patients, and different levels of depression should receive different mental health services (11, 12). Reasonable treatment measures can effectively alleviate the symptoms of depression; otherwise, lack of appropriate treatment may worsen the patient's state (13). In our previous research (14), we found that many Chinese college students did not have an ideal mental health literacy, and these students could not correctly judge depression and were reluctant to seek professional psychological help.

Students with severe depression should receive timely treatment to reduce their depressive symptoms; otherwise, some of them may decide to commit suicide (15). Some recent studies have suggested that the impact of the epidemic are profound and lasting, possibly leading to higher suicide rates among the population (16, 17), and a survey has shown that the suicide intention of the Chinese population is higher than that in normal times; especially, people aged 18–24 years (college students are in this age range) have a much higher suicide intention during the epidemic (18).

Therefore, this study conducted a meta-analysis of the incidence of depression among college students during the COVID-19 pandemic in China. In this regard, the proportions of mild, moderate, and severe depression among college students were calculated to help authorities provide targeted interventions to college students with different degrees of depression. In addition, a quantitative assessment of the coping styles of college students with depression was conducted, and the role of new information platforms, such as Weibo and mobile application services (APPs), in disseminating knowledge about the prevention and control of the COVID-19 pandemic as well as mental health among college students was evaluated.

## Methods

### Search Strategy

Six electronic databases (Web of Science, PubMed, Embase, WanFang, CNKI, and WeiPu) were searched for related studies published not later than July 2020. Furthermore, studies published only in English or Chinese were considered. The search terms included “COVID” OR “COVID19” OR “Coronavirus” OR “SARSCOV2” AND “college students” OR “university students” AND “depression” in the title and/or abstract.

### Study Selection

The included studies met the following criteria: (1) they investigated Chinese college students; (2) they were conducted during the COVID-19 outbreak; (3) they examined the emotional or psychological changes in college students; (4) they used valid diagnostic criteria for depression symptoms; (5) they were written in Chinese or English language; (6) they contained the necessary research outcomes needed for this study; (7) depression in this study refers to individuals showing obvious negative emotions such as decreased interest, hopelessness, inferiority, etc. These negative emotions can be evaluated using professional scales.

### Data Extraction

The following data from eligible studies were independently extracted by two authors: first author, year of publication, study design, research location, sample size, number of college students with varying degrees of depression, assessment tools for depression, coping styles of depressed college students, number of college students who obtained relevant information through electronic social media platforms, and other information.

### Outcome

The main outcome variable for this study was the prevalence of mild, moderate, and severe depression among Chinese college students during the COVID-19 pandemic.

Mild depression was defined as not being interested in many things. In this case, negative emotions do not affect normal work and study. These symptoms last no more than 2 weeks and can be alleviated by talking to family or friends (11, 12).

Moderate depression was defined as negative emotions such as pessimism, low productivity, and inability to fully engage in work and study. These symptoms last more than 2 weeks.

Severe depression was defined as a chronic lack of sleep, suicidal tendencies, and inability to work or study properly. For the classification of depression, this study referred to the International Classification of Diseases (ICD-10); relevant literatures were also reviewed, and the classification was consistent (11, 12).

The secondary objective of this study was to assess the role of new information platforms, such as Weibo and mobile APPs, in disseminating knowledge about the prevention and control of the COVID-19 pandemic as well as mental health among college students.

A mobile APP in this study refers to any mental health APP supported by iPhone and Android systems. Weibo is also called as Microblog, which is a platform based on user relationship information sharing, dissemination, and acquisition; users can form personal communities through various clients, such as Web.

### Risk of Bias Assessment

The risk of bias was assessed according to the guidelines of the Cochrane reviews (19). Two authors evaluated the following information: representativeness of sample, consistency of the survey tools, and information integrity. The included studies were graded according to the Newcastle Ottawa Scale, with respect to the above information.

### Statistical Analysis

The untransformed proportions (PRAW), log transformation (PLN), logit transformation (PLOGIT), arcsine transformation (PAS), and the Freeman–Tukey double arcsine transformation (PFT) were used to evaluate whether the distribution of the main outcome (rate of depression) conforms to a normal distribution (20). The index that was closest to the normal distribution was selected to perform rate merging. The rate of depression and the corresponding 95% confidence intervals (CIs) were calculated. Heterogeneity was assessed using the *I*^2^-test. Accordingly, an *I*^2^ > 50% indicated the existence of heterogeneity, and in this case a random model was adopted, whereas an *I*^2^ <50% implied low heterogeneity and, hence, a fixed model is adopted (21). In addition, publication bias was evaluated by a funnel plot and confirmed using Egger's test. All statistical analyses were conducted using R version 3.4.4 (R Project for Statistical Computing, Vienna, Austria). Statistical tests were considered significant when *P* < 0.05.

## Results

### Study Selection

**Figure 1:**
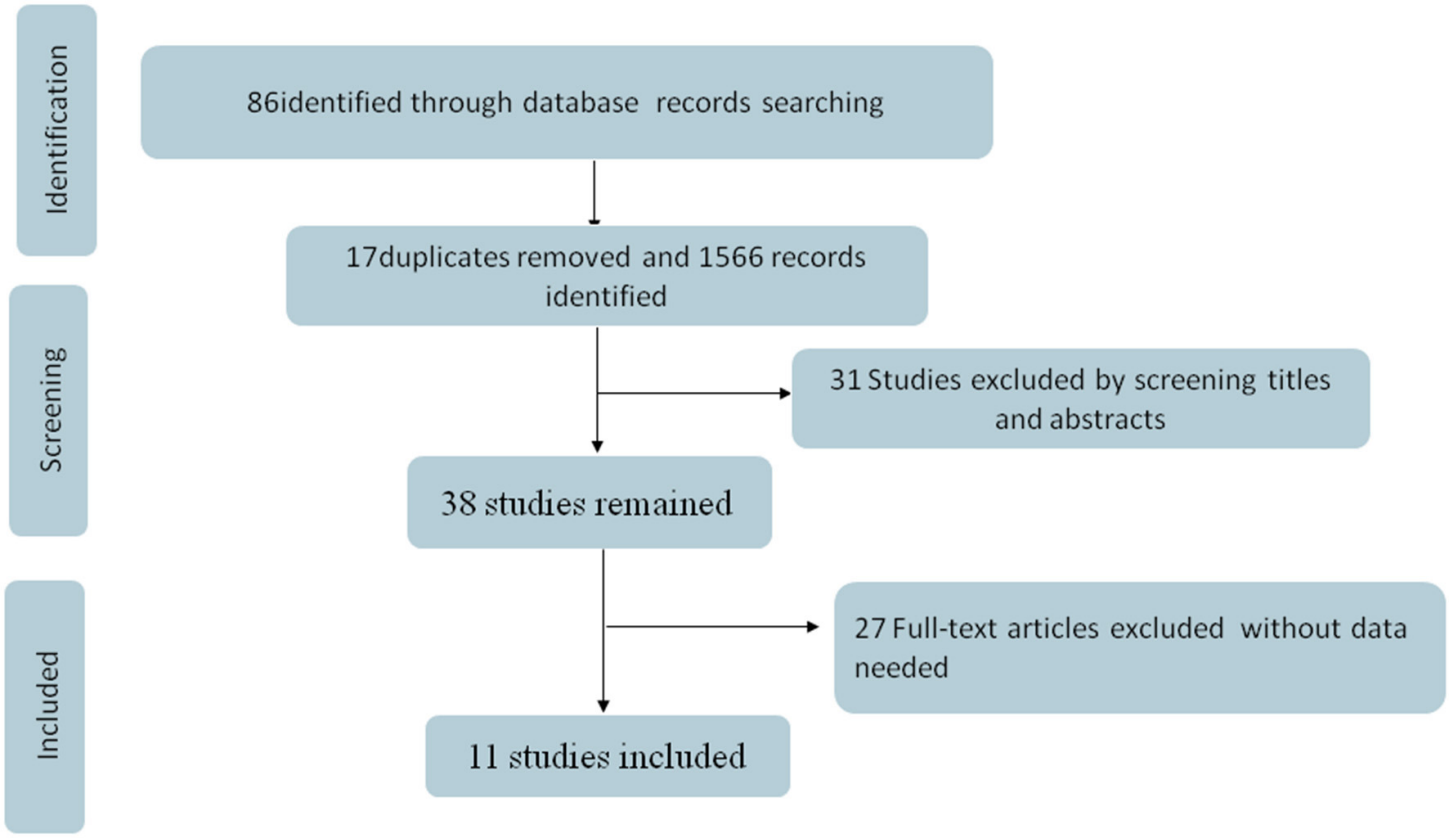
Flowchart presenting the steps of the literature search and selection.

We first obtained 86 related studies from six electronic databases (Web of Science, PubMed, Embase, WanFang, CNKI, and WeiPu). Of these, 17 were duplicates and so were removed. After screening the titles and abstracts of the remaining studies, 31 were excluded. Furthermore, among the 38 full-text studies left, 27 were ruled out because they did not have the outcomes of interest for this study. Finally, a total of 11 studies (22–32) with 25,020 Chinese college students were included in the present study. The flowchart is schematically shown in Figure 1.

### Study Characteristics

Detailed information about the included studies is shown in Table 1. Ten of the included studies were published in Chinese journals and one in an English journal. In terms of the diagnostic criteria, six studies used the SCL-90 (Symptom Checklist 90) while four studies used PHQ-9 (Patient Health Questionnaire 9) to assess depression symptoms. Samples were selected from different regions of China. Furthermore, the quality of the included literatures is shown in Table 1. In this regard, according to the Newcastle Ottawa Scale, four papers were evaluated to have four points and seven papers have three points.

**Table 1:** Basic information and data of all the included studies in the meta-analysis.

**References**	**Region (city)**	**Sample (total)** **/M/F**	**Mil number** **/M/F**	**Mil/mod** **/ser**	**Study design**	**Screening questionnaire**	**Outcomes**	**Type of college**	**Newcastle Ottawa** ** scale** ** (points)**	**Classification of published journals**
Zhu and Li (22)	Wuhan	838/344/494	546/231/395	546/66/14	Cross-sectional study	Self-design questionnaire	1	University	3	Authorative journal
Ding and Hu (23)	Fujian	3,055/1,420/1,635	1330/596/734	1,330/1,039/303	Cross-sectional study	National Health Commission questionnaire	1, 3	University	4	Authorative journal
Wei (24)	Guangzhou	6,289/–/–	1,013/–/–	1,013/222/75	Cross-sectional study	PHQ-9	1, 2	University	4	Unauthorative journal
Deng et al. (25)	Wuhan	517/135/382	15/5/10	15/2/1	Cross-sectional study	PHQ-9	1	University	3	Authorative journal
Liu (26)	Haerbing	553/292/261	89/44/45	89/53/44	Cross-sectional study	SCL-90	1	College	3	Authorative journal
Wang et al. (27)	Haerbing	1,111/203/908	279/49/230	279/–/24	Cross-sectional study	SCL-90	1, 3	University	4	Authorative journal
Chang et al. (28)	Guangdong	3,881/1,434/2,447	659/229/430	659/123/39	Cross-sectional study	PHQ-9	1, 2	University	4	Authorative journal
Ma et al. (29)	Shanxi	516/–/–	143/–/–	143/43/5	Cross-sectional study	SCL-90	1	College	3	Authorative journal
Cao et al. (30)	Shanxi	7,143/2,168/4,975	1518/525/993	1518/196/62	Cross-sectional study	SCL-90	1	University	3	Authorative journal
Mo (31)	Anhui	786/–/–	158/–/–	158/–/–	Cross-sectional study	SCL-90	2	University	3	Authorative journal
Zhong and Xiong (32)	Chengdu	331/155/176	95/36/59	95/–/23	Cross-sectional study	SCL-90	1	University	3	Unauthorative journal

*For outcomes, 1: there was information about the prevalence of depression; 2: included information about using new information platforms; 3: information about help seeking*.

*M, male; F, female; mil, mild depression; mod, moderate depression; ser, serious depression*.

### College Students With Mild Depressive Symptoms

**Figure 2:**
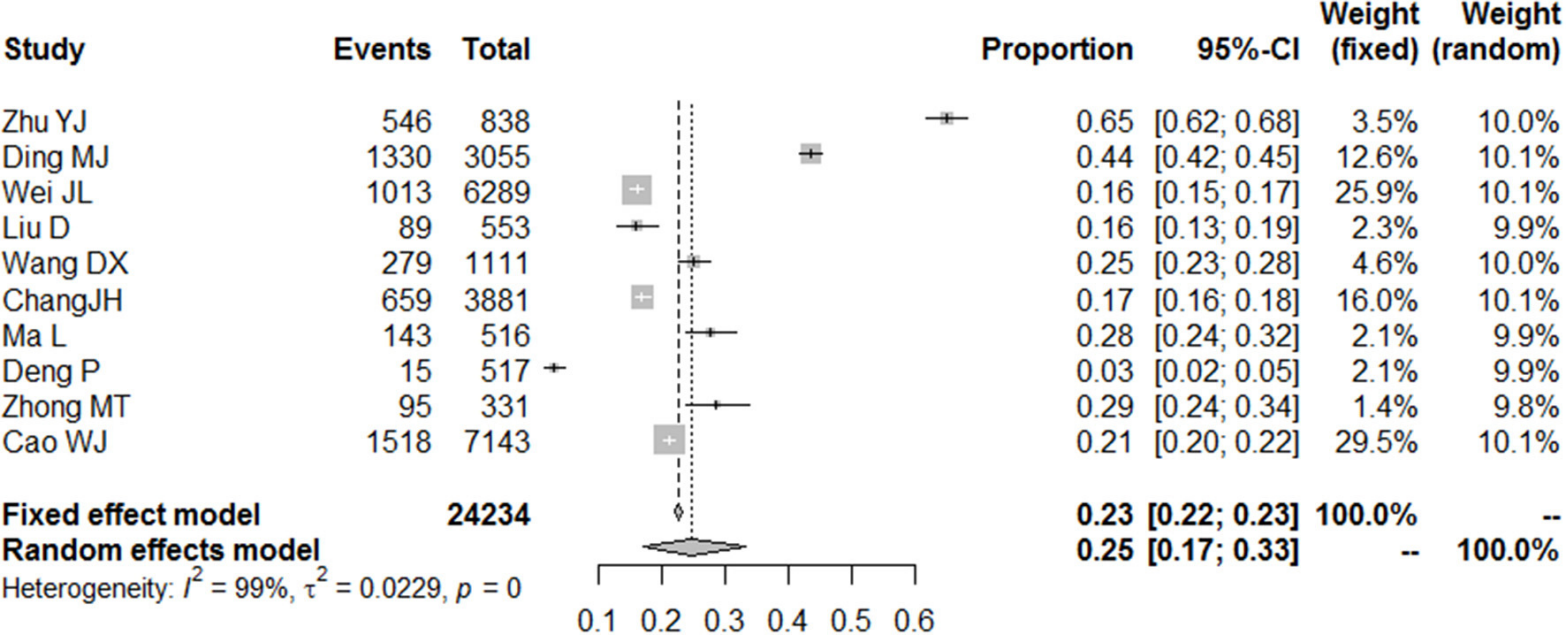
Forest plot of the incidence of mild depression among college students during COVID-19.

**Figure 3:**
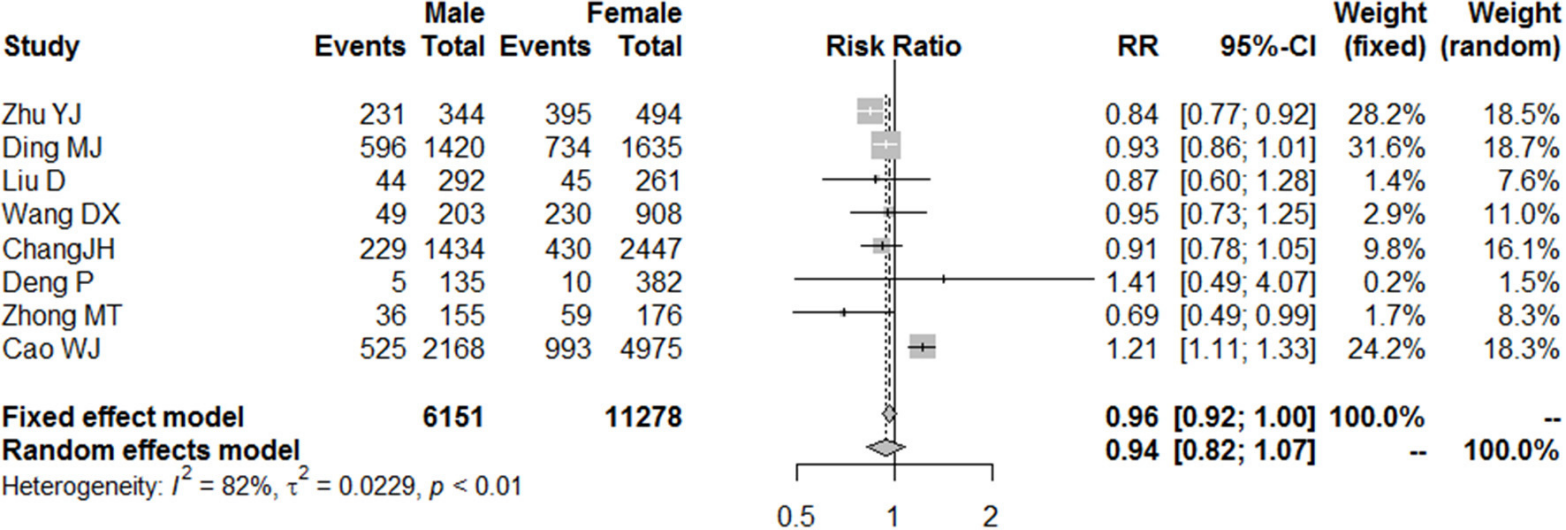
Forest plot of the incidence of mild depression between genders.

According to the diagnostic criteria for depression, college students with depression during the COVID-19 pandemic were classified as having mild, moderate, and severe depression (11). Additionally, 10 studies provided information on college students who suffered from mild depression during the COVID-19 pandemic, and the normality test indicated that logit conversion of the original rate was the closest to the normal distribution, so logit conversion was performed on the original rate before merging the rates. The result of heterogeneity indicated that there was significant heterogeneity in this result, so the random model was selected. During the COVID-19 pandemic, about 25% (95% CI = 17–33%) of college students suffered from mild depression (Figure 2). Based on the information in the included literature, the incidence of depression between the sexes was explored, and the results indicated that there was no significant difference between genders [relative risk (RR) = 0.94, 95% CI = 0.82–1.07]. Figure 3 shows the details.

### College Students With Moderate Depressive Symptoms

**Figure 4:**
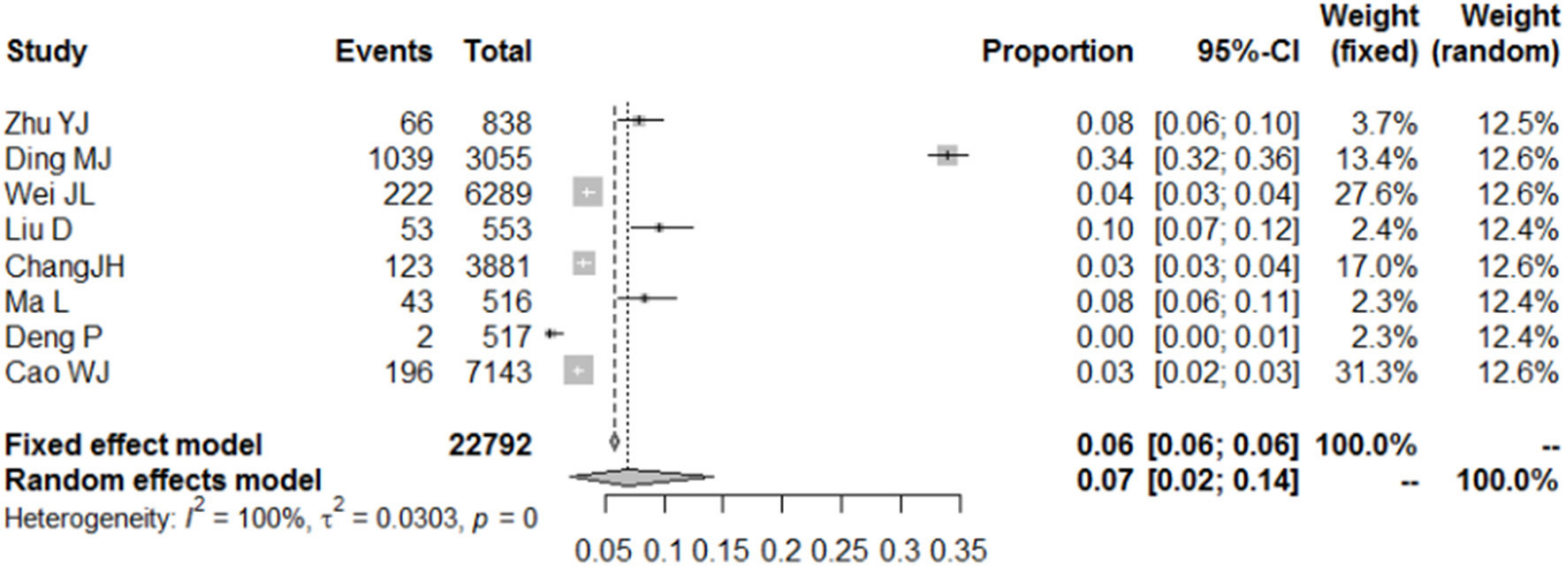
Forest plot of the incidence of mild depression among college students during COVID-19.

Eight included studies described the prevalence of moderate depression among college students during the COVID-19 pandemic, and this involved 5,000 subjects. The combined results, using a random model, showed that the proportion of college students suffering from moderate depression during the COVID-19 pandemic was 7% (95% CI = 2–14%) (Figure 4).

### College Students With Severe Depression

**Figure 5:**
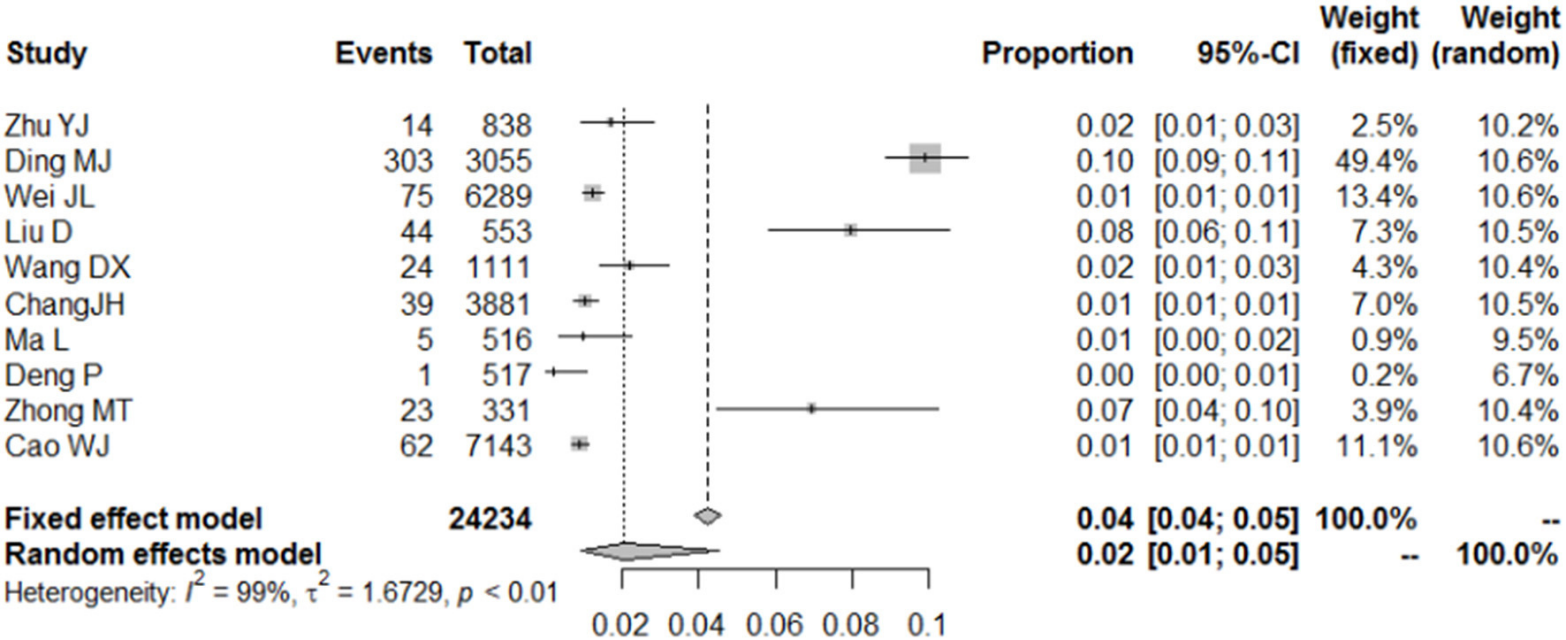
Forest plot of the incidence of mild depression among college students during COVID-19.

Furthermore, 10 different studies involving 24,234 college students found that 590 had major depression. The normality test indicated the use of the PFT to perform rate merging; due to significant heterogeneity, the random model was applied. During the COVID-19 pandemic, the combined incidence of severe depression among Chinese college students was 2% (95% CI = 1–5%). Figure 5 shows the details.

### Analysis of Channels Used by College Students to Acquire COVID-19 Knowledge

**Figure 6:**
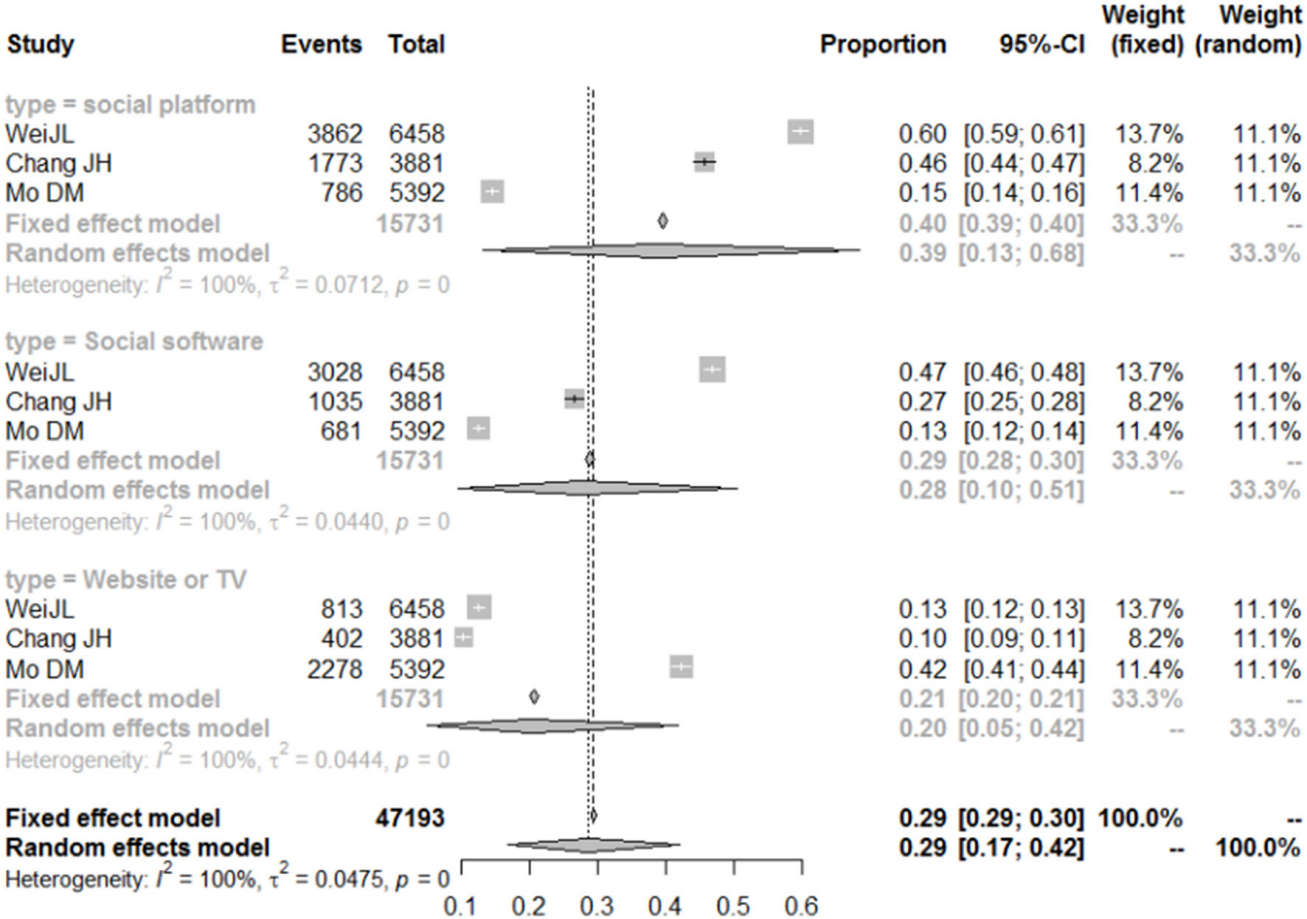
Forest plot of the importance of different information platforms in disseminating COVID-19 knowledge.

The proportion of college students who use WeChat and Weibo to acquire COVID-19 knowledge was 39% (95% CI = 13–68%). Social software (including mobile APPs and public accounts) also played an important role in spreading knowledge of the prevention and control of the pandemic. Our research results showed that about 28% (95% CI = 10–51%) of the college students acquired COVID-19 prevention and control knowledge through the foregoing channel. This proportion is slightly lower than that for social platform users, but higher than that for traditional communication channel users (Figure 6).

### Analysis on the Ways of Seeking Help for Depressed College Students

During the COVID-19 pandemic, college students in China were in social isolation. Research on college students' coping or seeking help for depression during the isolation period is beneficial for improving the quality of the mental health service system. The results indicated that 70% (95% CI = 51–85%) of the college students sought help from family members when they were depressed.

**Figure 7:**
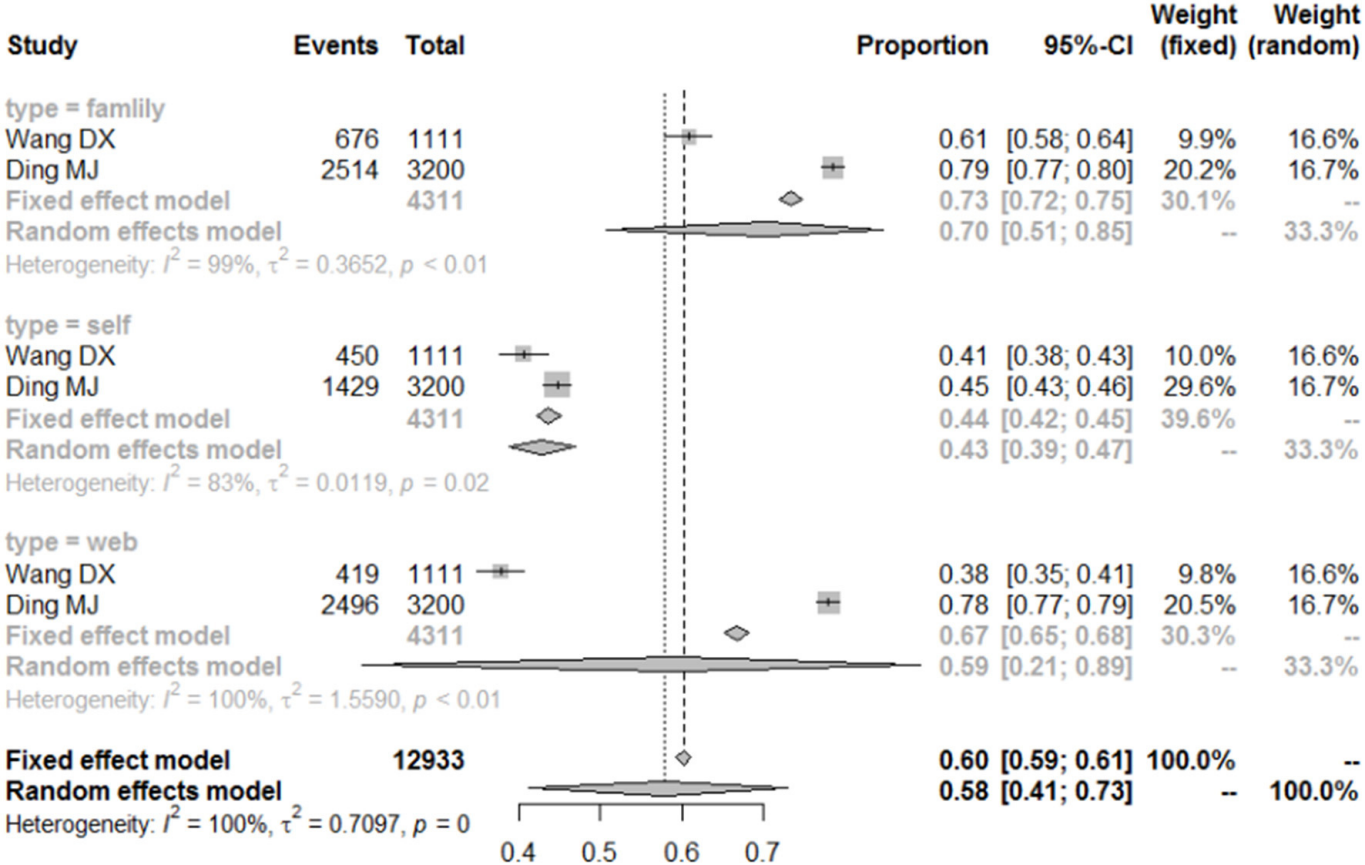
Forest plot of the coping styles of depressed college students.

Moreover, 59% (95% CI = 41–73%) of depressed college students often used mobile phone mental health APPs for help in dealing with depression. Figure 7 shows the details.

### Publication Bias

**Figure 8:**
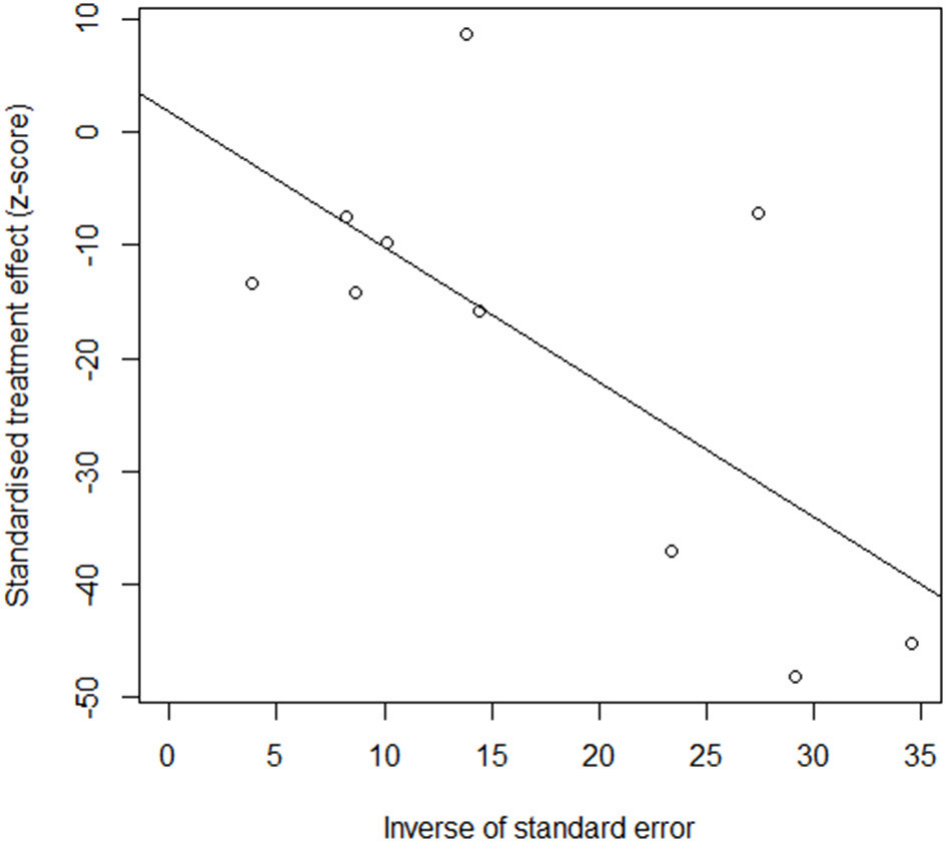
Funnel plot of publication bias.

The funnel plot is shown in Figure 8. The results of the Eggers test indicated that there was no significant publication bias (*t* = 0.51, *p* = 0.616, bias = 1.56, se.bias = 3.06, slope = 0.51), thus justifying the validity and credibility of this meta-analysis.

## Discussion

### Principal Findings

College students are in a special period of transition from teenagers to adults and have poor ability to adjust and cope with emergencies. Major public events can have negative effects on the psychology of young people, such as SARS in 2003 (33) and the Wenchuan earthquake in 2008 (34). Recently, the COVID-19 pandemic has lasted longer than the preceding events, and social isolation measures have been stricter than those for SARS in 2003 (35). Public health emergencies are strongly stressful situations for individuals; under such circumstances, people will show a lot of abnormal psychology and behavior, such as anxiety, depression, sleep disorders, and physical discomfort (16). Social supports based on social networks can effectively mitigate the impact of the epidemic on affected people. However, due to the restrictions of relevant policies, the function of social networks has been weakened; thus, during the COVID-19 pandemic, some people may commit suicide because they could not stand the strict social isolation policies or due to other reasons (32). A recent study indicated that people aged 18–24 years may have a higher risk of depression and a higher rate of suicidal thoughts than on normal days (18).

The COVID-19 outbreak in China came at a time when college students were in the Spring Festival holiday, and they had to isolate themselves at home for the next 6 months after the outbreak. Previous studies found that the strict social segregation policy may have a negative impact on children's psychology (36), suggesting that revising the policy so as to include psychological intervention measures was necessary.

This study explored the psychological state of college students during lockdown in the COVID-19 pandemic. The results indicated that college students suffered from depression at a higher rate than in normal circumstances, which was consistent with the concerns of some scholars (34, 37).

Specifically, the results of the meta-analysis showed that the proportion of college students with mild depression was 25% (95% CI = 17–33%), those with moderate depression was 7% (95% CI = 2–14%), and those with severe depression was 2% (95% CI = 1–5%). There was no significant gender difference in the incidence of mild depression (RR = 0.94, 95% CI = 0.82–1.07); however, this result may be related to the samples of the included literatures, in which gender parity was not considered when conducting the surveys.

Due to the non-uniform assessment criteria for depression used in the included literatures (there were four assessment criteria, including two questionnaires and two international depression assessment scales), the confidence intervals of the data obtained in this study were relatively wide. However, the results indicated that the COVID-19 pandemic has had a significant negative impact on the mental state of college students. Therefore, in order to reduce the impact of COVID-19 on the mental health of college students, colleges and universities should provide effective mental health services to their students during the COVID-19 pandemic.

Some scholars have conducted studies on the impact of COVID-19 on people's mental state, which recommended that adolescents, including university students, should be provided with effective mental health services to reduce the impact of the epidemic on them (38, 39).

In this study, college students with depression were classified as having mild, moderate, and severe depression according to the diagnostic criteria for depression, suggesting that different interventions should be developed according to the degree of depression in students in order to prevent the aggravation of depressive symptoms.

Thus, although previous studies showed that the COVID-19 pandemic negatively impacted on the healthcare systems in many countries, including mental health services^1^, it is imperative for college administrators to pay more attention to the mental health of their students in the same way as they do when protecting them from COVID-19.

With regard to help seeking for depression, the results of this study indicated that most depressed students sought help from their families, suggesting that social support, such as family, can play an important role in alleviating depression. Furthermore, about 59% of the depressed college students used mobile phone APPs of mental health category for help in dealing with bad emotions, indicating that these APPs also play an important role in helping depressed college students to cope with their bad emotions. However, the antidepressant effects of these mobile phone APPs were not evaluated in this study. Thus, an evaluation of the effectiveness of these mobile phone APPs in providing mental health services to Chinese college students should be conducted precisely because, although previous studies have confirmed that these APPs may play a role in providing mental health services for early depression, they also have some shortfalls (40). For example, some studies found that some psychological intervention APPs have disadvantages such as excessive disclosure of personal privacy, inappropriate use, and lack of professional psychological intervention content (40). Also, a study examining the effectiveness of a smartphone APP in treating depression found that the exact contribution of the APP in decreasing the depression scores was unclear (41).

In addition, concerning the sources of knowledge for the COVID-19 pandemic, this study found that Weibo, official accounts, and other social media platforms were not only the most important sources of knowledge about the COVID-19 pandemic accessed by Chinese college students but they also played an important role in communication. These results are consistent with the results of related previous studies.

### Limitations

Some limitations should be noted in this study. Firstly, most of the included studies investigated Chinese college students, which may preclude generalizing these results to other non-Chinese college students. Secondly, symptoms of depression were not a predefined outcome, hence may not have been accurately evaluated. Besides, among the included studies, there were different evaluation scales for depression, including PHQ-9, SCL-90, and the National Health Commission questionnaire, which may account for the heterogeneity in the results. Despite the preceding limitations, the present study provides valuable information for psychological interventions aimed at effectively improving the depression symptoms of college students.

### Conclusion

The prevalence of depression among college students in China was high during the lockdown in the COVID-19 pandemic. Thus, considering the adverse outcomes of depression, it is imperative that college administrators frequently screen college students in China for depression during the CIVID-19 pandemic and provide them with necessary psychological interventions to control and prevent depression. Social media platforms, such as WeChat and Weibo, and mental health APPs could provide an opportunity for psychological health information dissemination for college students in China. However, their effectiveness in reducing depression will have to be assessed.

## Author Contributions

SG and JX conceived and designed the analysis and performed the analysis. SG, JX, and AK wrote the paper. All authors contributed to the article and approved the submitted version.

## Conflict of Interest

The authors declare that the research was conducted in the absence of any commercial or financial relationships that could be construed as a potential conflict of interest.

## Footnotes

^1^Available online at: https://www.who.int/publications/i/item/WHO-2019-nCoV-EHS_continuity-survey-2020.1

## Abbreviations

COVID-19: 2019 novel coronavirus disease
APPs: application service
PHQ-9: Patient Health Questionnaire 9
SCL-90: Symptom Checklist 90.
